# HLA‐G in Hematological Malignancies: Immunoregulatory Mechanisms and Implications for Immune Evasion and Immunotherapy

**DOI:** 10.1155/jimr/8747974

**Published:** 2026-05-04

**Authors:** Thaís Lohana Chanel Pereira Ribeiro, Glenda Menezes Nogueira, Mateus Souza-Barros, Júlia Santos Moraes, Larissa Silva Santos, Lucas Silva Oliveira, Juniel Assis Crespo-Neto, Julia Goes Souza Ghedini, Fábio Magalhães-Gama, Adriana Malheiro, Fabíola Silva Alves-Hanna, Allyson Guimarães Costa

**Affiliations:** ^1^ Department of Teaching and Research, Fundação Hospitalar de Hematologia e Hemoterapia do Amazonas (HEMOAM), Manaus, Brazil, hemoam.am.gov.br; ^2^ Graduate Program in Applied Sciences in Hematology, Universidade do Estado do Amazonas (UEA), Manaus, Brazil, uea.edu.br; ^3^ Graduate Program in Basic and Applied Immunology, Institute of Biological Sciences, Universidade Federal do Amazonas (UFAM), Manaus, Brazil, ufam.edu.br; ^4^ Graduate Program in Biotechnology, Universidade Federal do Amazonas (UFAM), Manaus, Brazil, ufam.edu.br; ^5^ Graduate Program in Health Sciences, René Rachou Institute – Fundação Oswaldo Cruz (FIOCRUZ) Minas, Belo Horizonte, Brazil, cpqrr.fiocruz.br

**Keywords:** hematological malignancies, HLA-G, immune surveillance, immunotherapy, tumor immune evasion

## Abstract

Human leukocyte antigen G (HLA‐G) is a nonclassical major histocompatibility complex class I molecule whose increased expression has been consistently associated with unfavorable prognosis in solid tumors and has emerged as a potential immunotherapeutic target in hematological malignancies. HLA‐G exhibits limited genetic diversity and generates multiple isoforms that play a critical role in immune tolerance, being physiologically expressed in immunoprivileged tissues and aberrantly upregulated in a variety of pathological conditions, including cancer. Accumulating evidence indicates that elevated HLA‐G expression contributes to tumor immune evasion and influences clinical outcomes in patients with leukemia, lymphoma, and multiple myeloma. Genetic variability within the HLA‐G gene, particularly polymorphisms located in regulatory regions such as the 14‐base pair insertion/deletion, has been associated with cancer susceptibility, disease progression, and adverse prognosis. In hematological malignancies, specific genotypes, including the homozygous deletion variant, have been linked to increased levels of membrane‐bound and soluble HLA‐G, correlating with impaired immune surveillance and reduced survival, particularly in chronic lymphocytic leukemia. Moreover, HLA‐G expressions may be modulated by inflammatory cytokines, such as interferon‐γ, further shaping the immunosuppressive tumor microenvironment. By functioning as a nonclassical immune checkpoint, HLA‐G represents a promising target for innovative immunotherapeutic strategies, including immune checkpoint blockade combinations and chimeric antigen receptor (CAR)‐T cell approaches directed against HLA‐G‐expressing malignant cells. In this review, we summarize current knowledge regarding HLA‐G expression, genetic polymorphisms, and immunoregulatory mechanisms in hematological malignancies, highlighting their clinical and translational implications. Improved understanding of HLA‐G‐mediated immune modulation may contribute to the development of novel prognostic biomarkers and therapeutic strategies aimed at restoring effective antitumor immunity.

## 1. Introduction

Hematological neoplasms comprise a heterogeneous group of malignancies commonly referred to as liquid tumors, characterized by the proliferation of cancerous cells of hematopoietic origin within the blood, bone marrow, and lymphoid tissues. This group includes three major entities: leukemias, lymphomas, and multiple myeloma (MM) [1]. Leukemias are defined by the clonal expansion of malignant hematopoietic cells in the bloodstream and hematopoietic organs and are classified according to immunophenotypic, cytogenetic, molecular, and clinical features. Based on lineage and disease progression, leukemias are broadly categorized as lymphoid or myeloid and as acute or chronic, with acute lymphoblastic leukemia (ALL), acute myeloid leukemia (AML), chronic lymphocytic leukemia (CLL), and chronic myeloid leukemia (CML) representing the most prevalent subtypes [2].

Lymphomas arise from malignant transformation of lymphoid cells within the lymphatic system and are traditionally divided into Hodgkin lymphoma (HL) and non‐Hodgkin lymphoma (NHL). HL is characterized by the presence of neoplastic Reed‐Sternberg cells derived mainly from B lymphocytes and develops within a highly immunologically active microenvironment. Despite its origin in lymph nodes, HL may disseminate to extranodal sites, including the spleen and bone marrow. It is further classified into nodular sclerosis, mixed cellularity, lymphocyte‐rich, and lymphocyte‐depleted subtypes, with Epstein–Barr virus (EBV) infection contributing to tumorigenesis in approximately half of the cases [3,4].

In contrast, NHL encompasses a broad spectrum of more than 40 biologically and clinically distinct entities, ranging from indolent to highly aggressive forms. Among these, follicular lymphoma and diffuse large B‐cell lymphoma (DLBCL) represent the most common indolent and aggressive subtypes, respectively. NHL pathogenesis is driven by diverse genetic alterations, including chromosomal translocations and somatic mutations, and may be influenced by infectious agents such as EBV, hepatitis B virus (HBV), and human immunodeficiency virus (HIV) [3, 4].

MM is characterized by the accumulation of malignant plasma cells in the bone marrow, where a permissive tumor microenvironment supports disease progression. Interactions between neoplastic plasma cells and nonmalignant stromal components promote the production of immunomodulatory cytokines, notably interleukin‐6 (IL‐6), which plays a central role in myeloma cell survival, immune escape, and therapeutic resistance [5, 6]. Similar to solid tumors, hematological malignancies exploit multiple mechanisms to evade immune surveillance, including impairment of cytotoxic lymphocyte activity and induction of immunosuppressive signaling pathways within the tumor microenvironment [7].

In this context, human leukocyte antigen G (HLA‐G), a nonclassical major histocompatibility complex class I molecule with potent immunoregulatory properties, has emerged as a relevant mediator of tumor immune evasion. Aberrant expression of HLA‐G by malignant cells contributes to immunosuppression through the inhibition of NK cells, cytotoxic T lymphocytes (CTLs), and antigen‐presenting cells (APCs), thereby favoring tumor progression [8]. Increased HLA‐G expression has been consistently associated with unfavorable clinical outcomes in several solid tumors [9–11].

Importantly, genetic polymorphisms within the HLA‐G gene, particularly those affecting regulatory regions, have been linked to altered expression of membrane‐bound HLA‐G and increased circulating levels of soluble HLA‐G (sHLA‐G), further amplifying its immunosuppressive effects [12, 13]. Although accumulating evidence suggests that HLA‐G expression and genetic variability may influence disease progression and prognosis in hematological malignancies, its precise role across different hematopoietic cancers remains incompletely understood.

In this review, we critically summarize current evidence regarding the role of HLA‐G in hematological malignancies, with emphasis on its immunoregulatory mechanisms, genetic regulation, and clinical relevance. We also discuss the potential of HLA‐G and its genetic variants as prognostic biomarkers and therapeutic targets in the context of tumor immune evasion and immunotherapy.

## 2. HLA‐G in Blood Diseases

HLA‐G is a central mediator of immune tolerance and differs fundamentally from classical HLA class I molecules, which primarily promote immune activation. Through direct interaction with inhibitory receptors and indirect induction of suppressive immune cell populations, HLA‐G exerts potent inhibitory effects on CD4^+^ and CD8^+^ T cells, natural killer (NK) cells, dendritic cells, and macrophages. Beyond immune inhibition, HLA‐G influences angiogenesis, cell migration, and tissue remodeling, playing a pivotal role in physiological tolerance during pregnancy, particularly in placental development and maternal–fetal immune balance [14–16].

Given its immunomodulatory properties, HLA‐G has been extensively investigated in a wide range of pathological conditions, including transplantation, autoimmune disorders, infectious diseases, and cancer. In hematological disorders, accumulating evidence indicates that aberrant expression of HLA‐G, particularly in its soluble form, contributes to immune dysregulation and disease progression. Notably, in hematological malignancies, soluble HLA‐G (sHLA‐G) appears to be more frequently detected than membrane‐bound isoforms, suggesting a preferential role for systemic immunosuppression rather than localized immune inhibition [17].

In lymphoproliferative disorders, high levels of HLA‐G transcripts have been reported in the absence of detectable surface expression, reinforcing the relevance of the soluble isoform. Elevated sHLA‐G concentrations have been observed in ~70% of patients with chronic lymphocytic leukemia (CLL), 53% of those with B‐cell NHL, and 45% of patients with T‐cell NHL, even when membrane‐bound HLA‐G expression is absent [17]. These findings support the concept that sHLA‐G may act as a dominant immunosuppressive mediator in the hematological tumor microenvironment.

Beyond malignant conditions, dysregulated HLA‐G expression has also been implicated in non‐neoplastic hematological diseases with an immune‐mediated component. In acquired aplastic anemia (AA), an autoimmune disorder characterized by immune‐mediated destruction of hematopoietic stem and progenitor cells, increased sHLA‐G levels have been associated with impaired B‐cell development. Sun et al. demonstrated that elevated HLA‐G and ILT2 expression may contribute to excessive immune suppression within the bone marrow, suggesting that therapeutic blockade of the HLA‐G–ILT2 axis could restore B‐cell proliferation and hematopoietic balance [18].

Altered HLA‐G expression has also been reported in inherited and myeloproliferative blood disorders. In β‐thalassemia, a genetic hemoglobinopathy resulting from defective β‐globin synthesis, patients exhibit significantly higher circulating levels of HLA‐G compared with healthy controls, indicating a potential role for HLA‐G in chronic immune modulation associated with ineffective erythropoiesis [19]. Similarly, in polycythemia vera, a myeloproliferative neoplasm driven predominantly by the JAK2 V617F mutation, soluble HLA‐G has been shown to negatively regulate proliferative signaling pathways, potentially inducing cell cycle arrest at the G1 phase and attenuating excessive erythroid expansion [20–22]. These observations raise the possibility that sHLA‐G may serve as a modulatory factor influencing disease severity and therapeutic response.

Immune thrombocytopenic purpura (ITP) further exemplifies the immunoregulatory role of HLA‐G in hematological disorders. In this autoimmune condition, HLA‐G expression has been associated with suppression of pro‐inflammatory Th1 and Th17 responses and a shift toward a Th2‐biased cytokine profile, characterized by increased IL‐4 and IL‐10 production and reduced levels of tumor necrosis factor‐α, IL‐12, and IL‐17. Additionally, HLA‐G‐modulated immune cells have been shown to reduce platelet apoptosis, suggesting a protective role in disease pathophysiology [23].

Collectively, these findings highlight HLA‐G as a critical immunoregulatory molecule across a broad spectrum of hematological diseases. The predominance of soluble HLA‐G in many of these conditions underscores its systemic immunosuppressive capacity and supports ongoing interest in exploiting HLA‐G‐based strategies for therapeutic modulation. Indeed, experimental approaches evaluating the use of soluble HLA‐G or HLA‐G‐derived molecules as immunotherapeutic agents in blood and circulatory disorders, including anemia and ischemic conditions, suggest potential clinical benefits, although further translational and clinical studies are required to define their safety and efficacy [24].

## 3. The Role of HLA‐G in the Tumor Microenvironment

HLA‐G is a nonclassical major histocompatibility complex class I (MHC‐I) molecule characterized by limited genetic diversity and restricted tissue expression. Unlike classical HLA class I molecules, which primarily activate immune responses, HLA‐G plays a central role in immune tolerance. Its physiological relevance is best exemplified during pregnancy, where HLA‐G expression by trophoblastic cells contributes to maternal–fetal immune tolerance and successful placental development [8, 25]. Beyond gestation, constitutive HLA‐G expression has been described in immunoprivileged tissues, including the cornea, thymus, and endothelial and erythroid precursors, where it participates in fine‐tuning immune homeostasis under physiological and pathological conditions (28).

HLA‐G exerts its immunomodulatory effects through the generation of multiple isoforms derived from alternative splicing of its primary transcript. Seven isoforms have been described, including four membrane‐bound (HLA‐G1, G2, G3, and G4) and three soluble forms (HLA‐G5, G6, and G7). Soluble HLA‐G isoforms are detectable in plasma, amniotic fluid, and umbilical cord blood of healthy individuals, supporting their role in systemic immune regulation [26]. Importantly, methodological aspects influence HLA‐G quantification; plasma‐based measurements are considered more reliable than serum‐based assays, as clot formation can retain HLA‐G molecules and lead to underestimation in serum samples [27].

Among the isoforms, membrane‐bound HLA‐G1 and soluble HLA‐G5 are the most extensively studied due to their structural similarity to classical HLA class I molecules and their capacity to interact with inhibitory immune receptors. These include leukocyte immunoglobulin‐like receptor B1 (LILRB1), expressed on B cells, T cells, natural killer (NK) cells, monocytes, and dendritic cells (DCs); leukocyte immunoglobulin‐like receptor B2 (LILRB2), predominantly expressed on myeloid cells; and killer cell immunoglobulin‐like receptor 2DL4 (KIR2DL4), a receptor selectively expressed by the CD56^+^ subset of NK cells [28]. Engagement of these receptors by HLA‐G results in profound inhibition of NK cell cytotoxicity, suppression of T‐cell and NK‐cell proliferation, impairment of DC maturation and antigen‐presenting capacity, and induction of regulatory and suppressor immune cell populations [29].

**Figure 1:**
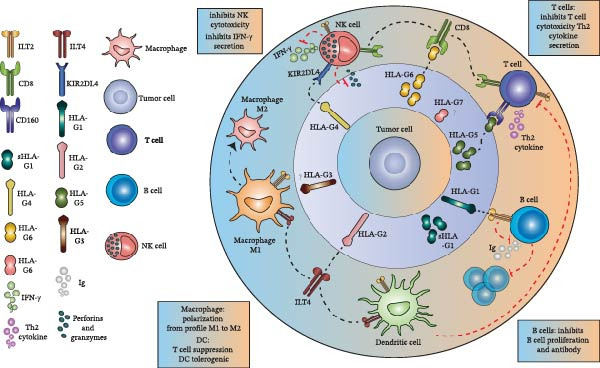
HLA‐G isoforms and their immunomodulatory roles in the tumor microenvironment. Human leukocyte antigen G (HLA‐G) isoforms exert potent immunoregulatory effects through interactions with inhibitory receptors expressed on multiple immune cell populations. The membrane‐bound and soluble HLA‐G1 isoforms interact with leukocyte immunoglobulin‐like transcript 2 (ILT2), resulting in inhibition of B‐cell antibody production and proliferation. In dendritic cells, HLA‐G–ILT2 engagement suppresses T‐cell activation and promotes a tolerogenic dendritic cell phenotype. HLA‐G4 interacts with the natural killer (NK) cell inhibitory receptor KIR2DL4, leading to reduced interferon‐gamma (IFN‐γ) secretion. In addition, the interaction between the soluble HLA‐G6 isoform and the CD8 receptor inhibits the cytolytic activity of NK cells and cytotoxic T lymphocytes, thereby contributing to immune evasion within the tumor microenvironment. Abbreviations: *ILT2*, immunoglobulin‐like transcript 2; *ILT4*, immunoglobulin‐like transcript 4; *DCs*, dendritic cells; *KIR2DL4*, killer cell immunoglobulin‐like receptor with two Ig domains and a long cytoplasmic tail 4; *IFN*‐γ, interferon‐gamma; *CD8*, cluster of differentiation 8.

The expression profile of HLA‐G isoforms varies according to cell type and pathological context. HLA‐G1 is the predominant isoform expressed on fetal cells and represents the only isoform consistently detected at the cell surface. HLA‐G2 may also be expressed by tumor cells and immune cells, whereas HLA‐G3 is rarely detected in malignant tissues. Soluble HLA‐G5 has been reported in several tumor types, while HLA‐G4 and G6 expression in cancer remains poorly documented, and HLA‐G7 is infrequently observed [30]. This heterogeneity underscores the complexity of HLA‐G‐mediated immune regulation within the tumor microenvironment (Figure 1).

Induction of HLA‐G expression, in either membrane‐bound or soluble form, has been described in a wide range of pathological conditions, including cancer, chronic inflammation, and allergic diseases [31]. In oncological settings, HLA‐G expression has been reported in more than 20 tumor types and is frequently associated with parameters of tumor aggressiveness, immune escape, and poor clinical outcome [32]. Given the inherent genetic instability of malignant cells, aberrant HLA‐G expression may emerge at different stages of tumor development, contributing dynamically to immune evasion during disease progression [33].

Elevated levels of HLA‐G have been reported across a wide range of malignancies, including hepatocellular carcinoma [34], prostate cancer and benign prostatic hyperplasia [35], glioma, and cutaneous melanoma, as well as hematopoietic and trophoblastic tumors [36–38]. In addition, increased circulating levels of sHLA‐G have been described in patients with melanoma, neuroblastoma, lymphoproliferative disorders, and breast, ovarian, and colorectal carcinomas, compared with healthy individuals or those with benign conditions [35, 36, 39, 40].

HLA‐G exerts a potent inhibitory effect on CD8^+^ CTLs, impairing their antigen‐specific cytolytic activity against infected or allogeneic targets. This effect has been demonstrated in functional assays in which HLA‐G1‐transfected cells inhibit peptide‐dependent lysis. Mechanistically, this suppression is mediated through interactions with inhibitory receptors such as ILT2 and ILT4, leading to the induction of apoptosis (e.g., via Fas/FasL pathways) and reduced production of cytotoxic mediators, including granzymes and perforin. These effects have been observed in both alloproliferative responses and virus‐specific cytotoxicity. Furthermore, in vitro studies indicate that HLA‐G expression on target cells or APCs can mediate immune inhibition via trogocytosis, thereby extending its suppressive effects beyond direct cell–cell interactions [41, 42].

In pathological contexts, such as viral infections and cancer, CD8^+^ T cells exposed to HLA‐G exhibit features of functional exhaustion, including reduced degranulation (CD107a) and decreased production of effector cytokines such as IFN‐γ, which have been associated with poorer clinical outcomes. The soluble isoform HLA‐G5 further amplifies this immunosuppressive effect by inducing senescence in NK cells and CTLs in an antigen‐independent manner, primarily through interactions with receptors such as KIR2DL4 and CD160. Notably, this inhibitory effect has been described as dose‐dependent and can be partially reversed by anti‐HLA‐G antibodies, highlighting its potential as a therapeutic target [43].

APCs expressing HLA‐G1, including monocytes and dendritic cells, exhibit impaired maturation, characterized by reduced expression of MHC class II molecules, CD80/CD86 co‐stimulatory markers, and diminished antigen‐presenting capacity. This results in suboptimal T‐cell activation and contributes to the establishment of a tolerogenic immune profile. Mechanistically, this process has been associated with cytokine‐mediated signaling pathways, including IL‐4‐induced activation of STAT3, promoting an M2‐like phenotype and the secretion of immunosuppressive mediators such as IL‐10 and TGF‐β. In transplantation settings, the presence of HLA‐G^+^ infiltrating APCs has been correlated with graft tolerance, particularly through the suppression of acute rejection responses [44].

Interactions between HLA‐G and its inhibitory receptors ILT2 and ILT4 on APCs impair key innate immune functions, including chemotaxis and phagocytosis, thereby facilitating tumor and viral immune escape. This has been observed in melanoma, where HLA‐G^+^ APCs contribute to the recruitment of myeloid‐derived suppressor cells (MDSCs). In addition, soluble HLA‐G can amplify these immunosuppressive effects through shedding and intercellular transfer mechanisms, including trogocytosis, thereby extending suppression to neighboring APCs. Notably, HLA‐G‐expressing APCs have been shown to induce suppressive CD4^+^ regulatory T cells (Tregs), further reinforcing immune tolerance [45].

At the molecular level, HLA‐G‐mediated immunoregulation primarily involves engagement of inhibitory receptors such as ILT2 (CD85j) and ILT4 (CD85d), which recruit phosphatases SHP‐1 and SHP‐2, leading to inhibition of TCR‐ and NK cell‐mediated signaling pathways. This results in cellular anergy and G0/G1 cell cycle arrest. Additionally, activation of the Fas/FasL pathway promotes apoptosis in activated CTLs, while KIR2DL4 signaling in NK cells has been associated with the induction of cellular senescence, partly mediated by dysregulated IFN‐γ production. In APCs, ILT4 engagement has been linked to activation of the IL‐6/STAT3 axis and suppression of pro‐inflammatory NF‐κB signaling, further promoting a tolerogenic phenotype [46].

Moreover, lateral transfer of HLA‐G via trogocytosis or extracellular vesicles, including exosomes, contributes to the propagation of nonspecific immunosuppression by converting immunocompetent cells into suppressive phenotypes. Soluble isoforms such as HLA‐G5 and HLA‐G6 can bind to receptors including CD8 and CD160, inducing apoptosis in CD8^+^ T cells and inhibiting B‐cell proliferation. Finally, crosstalk with additional immunosuppressive pathways, such as IDO and PD‐L1, may further amplify these effects, particularly in chronic inflammatory and tumor‐associated contexts [47].

The bone marrow microenvironment represents a highly specialized and dynamic niche that regulates hematopoietic stem cell (HSC) homeostasis through interactions with stromal, endothelial, and osteoblastic cells, as well as components of the extracellular matrix (ECM). Cellular elements such as CXCL12‐abundant reticular (CAR) cells, macrophages, and megakaryocytes provide key regulatory signals, including CXCL12 and stem cell factor (SCF), which maintain HSC quiescence and self‐renewal within perivascular and endosteal niches. Under physiological conditions, this tightly regulated balance prevents stem cell exhaustion and ensures continuous hematopoiesis [48].

In hematological malignancies, such as AML and CML, this microenvironment is profoundly remodeled to support malignant clones. Leukemic blasts can downregulate stromal CXCL12 expression via HIF‐1 α signaling, disrupt adhesion mediated by integrins such as VLA‐4 and VLA‐5, and promote the recruitment of immunosuppressive macrophages that secrete cytokines including IL‐6 and TGF‐β. These alterations contribute to enhanced leukemic cell survival, niche adaptation, and resistance to therapies such as imatinib. Furthermore, studies have demonstrated clonal heterogeneity within the bone marrow niche, with extracellular matrix components, such as collagen IV and fibronectin, facilitating leukemic cell migration to the peripheral circulation and contributing to disease relapse [49, 50].

Experimental and clinical evidence supports a direct role for HLA‐G in shaping an immunosuppressive tumor microenvironment. Mizuno et al. demonstrated that interferon‐γ stimulation induces HLA‐G expression in AML, with a subset of primary leukemic cells showing marked upregulation following cytokine exposure [51]. Similarly, Nückel et al. reported HLA‐G expression in B‐cell chronic lymphocytic leukemia (B‐CLL), where higher proportions of HLA‐G‐positive leukemic cells were associated with shorter progression‐free survival, outperforming conventional prognostic markers such as ZAP‐70 and CD38 [52]. In this context, elevated plasma levels of interleukin‐10 and soluble HLA‐G further support the link between HLA‐G expression and systemic immunosuppression in B‐CLL.

Additional studies have confirmed the expression of membrane‐bound HLA‐G in leukemic blasts. Flow cytometric analyses of AML samples revealed high HLA‐G1 expression in CD45^dim blast populations, suggesting that leukemic cells may directly exploit HLA‐G‐mediated immune inhibition [53]. More recently, Alkhouly et al. demonstrated that cytokines such as IL‐10 and IFN‐γ regulate HLA‐G expression in ALL, while increased HLA‐G expression on NK cells at diagnosis was associated with impaired immune effector function (57). Collectively, these findings position HLA‐G as a dynamic, cytokine‐responsive regulator of the hematological tumor microenvironment, influencing both malignant and immune cell behavior.

## 4. Polymorphisms in the HLA‐G Gene and HLA‐G Molecules

### 4.1. Leukemias

**Figure 2:**
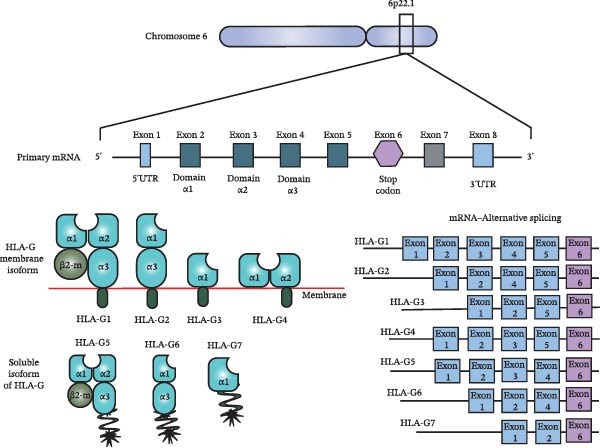
Genomic organization of the HLA‐G gene and generation of transcript isoforms by alternative splicing. Schematic representation of the HLA‐G gene locus, its primary messenger RNA (mRNA), and the distinct mRNA transcripts generated through alternative splicing, giving rise to membrane‐bound and soluble HLA‐G isoforms. Abbreviations: 5^′^ *UTR*, 5^′^ untranslated region; 3^′^ *UTR*, 3^′^ untranslated region.

The HLA‐G gene is a nonclassical major histocompatibility complex class I (MHC‐I) gene encoding an α‐chain composed of up to three extracellular domains that are not covalently associated with β2‐microglobulin. The HLA‐G gene is located on chromosome 6p22.1 and generates multiple isoforms through alternative mRNA splicing. To date, seven isoforms have been described, including four membrane‐bound forms (HLA‐G1 to HLA‐G4) and three soluble isoforms (HLA‐G5 to HLA‐G7) (Figure 2) [54, 55].

Compared with classical HLA class I genes, HLA‐G exhibits limited polymorphism. Its locus comprises seven introns and eight exons, with a premature stop codon located in exon 6. Despite this restricted variability, functionally relevant polymorphisms have been identified in intronic regions, the promoter, and particularly within the 3^′^ untranslated region (3^′^UTR). It is estimated that ~53 alleles, 18 full‐length proteins, and two null alleles have been described to date [54, 56]. These genetic variations are known to influence transcriptional regulation, mRNA stability, and post‐transcriptional control of HLA‐G expression.

Over the past decades, numerous studies have investigated polymorphisms in the HLA‐G gene and their association with autoimmune, inflammatory, infectious diseases, and cancer. Among these, the 14‐base pair (bp) insertion/deletion polymorphism located in exon 8 of the 3^′^UTR is the most extensively characterized. This polymorphism has frequently been associated with increased cancer susceptibility, unfavorable clinical outcomes, and reduced survival in oncological patients [57, 58].

In patients diagnosed with CLL, the homozygous del/del genotype of the 14 bp polymorphism has been associated with increased expression of membrane‐bound HLA‐G compared with ins/del and ins/ins genotypes. Consistently, significantly higher concentrations of sHLA‐G were observed in individuals carrying the del/del genotype, reinforcing the functional relevance of this variant in regulating HLA‐G expression [13]. Similarly, in CML, specific HLA‐G alleles have been shown to modulate immune responses and clinical outcomes. Increased sHLA‐G levels were observed in patients carrying the homozygous alleles G01:01: 01 or G01:01: 02 compared with G^∗^01:01: 03, which was more frequently detected in patients achieving an earlier molecular response and longer event‐free survival [56].

In the context of AML, susceptibility analyses revealed a significantly increased frequency of the HLA‐G UTR‐3 haplotype (*14 bp deletion*, *+3003T*, *+3010C*, *+3027C*, *+3035C*, *+3142G*, *+3187A*, *and +3196 C*) in patients compared with healthy donors. Moreover, membrane‐bound HLA‐G expression was detected on leukemic blasts, as well as on DC‐10 cells and CD4^+^ regulatory T cells, suggesting that both malignant and immunoregulatory cells may cooperate to establish an immunosuppressive microenvironment favoring leukemic immune escape [53].

In the setting of hematopoietic stem cell transplantation (HSCT), Chen et al. (2023) reported that single nucleotide polymorphisms (SNPs) located in the 3UTR (*rs371194629* and *rs9380142*) and a UTR‐3 haplotype, as well as SNPs in the 5UTR (*rs3823321* and *rs1736934*) and the G0104a haplotype, were associated with the development of graft‐versus‐host disease (GVHD). Although no direct associations were found with relapse or mortality, these polymorphisms may contribute to the regulation of post‐transplant immune tolerance and could serve as predictive markers for adverse outcomes following HSCT [59].

Elevated plasma concentrations of sHLA‐G have also been consistently reported in acute leukemias. Patients with ALL (*n* = 28) and AML (*n* = 47) exhibited significantly higher sHLA‐G levels compared with healthy controls (*p* <0.001 and *p* <0.01, respectively). Among AML subtypes, higher sHLA‐G concentrations were observed in FAB M4 and M5 compared with M1 and M2 subtypes. In ALL, patients with T‐ALL displayed increased sHLA‐G levels relative to those with B‐ALL. In vitro experiments further demonstrated increased HLA‐G mRNA expression in B‐ALL and T‐ALL cells following stimulation with immunomodulatory cytokines, including IL‐10, IFN, GM‐CSF, and IL‐2, highlighting the cytokine‐dependent regulation of HLA‐G expression in leukemic cells [60].

### 4.2. Lymphomas

The lymph node microenvironment is highly organized and functionally stratified into distinct compartments, including the cortex (containing primary and secondary B‐cell follicles with germinal centers), the paracortex (T‐cell zone enriched in dendritic cells and CD4^+^/CD8^+^ T cells), and the medulla (comprising cords with plasma cells and sinus‐associated macrophages). High endothelial venules (HEVs) and lymphatic sinusoids regulate the trafficking of lymphocytes and antigens through chemokine gradients, particularly involving CCR7 and CXCL13, thereby optimizing antigen presentation and adaptive immune activation [61].

In lymphomas, this microenvironment is frequently co‐opted to support tumor growth and immune evasion. For instance, in activated B‐cell‐like diffuse large B‐cell lymphoma (ABC‐DLBCL), malignant cells promote the expansion of immunosuppressive populations, including M2‐polarized tumor‐associated macrophages (CD163^+^ TAMs) and myeloid‐derived suppressor cells (MDSCs), largely through IL‐10/STAT3 signaling. These changes contribute to the suppression of CD8^+^ T‐cell activity, in part via PD‐L1‐mediated pathways. In classical Hodgkin lymphoma, Reed–Sternberg cells secrete chemokines such as CCL5, recruiting eosinophils and mast cells and promoting a pro‐angiogenic environment through increased VEGF production, which has been associated with poorer clinical outcomes in cases with high vascular density [62].

Therapeutic strategies targeting the lymphoma microenvironment have also emerged. For example, the Bruton’s tyrosine kinase (BTK) inhibitor ibrutinib has shown efficacy in mantle cell lymphoma (MCL) by disrupting tumor–stromal interactions and reducing microenvironmental support for malignant cells [62].

Evidence supporting the role of HLA‐G genetic variability and expression in lymphomas has progressively accumulated over the past two decades. In classical Hodgkin lymphoma (cHL), a study involving 20 patients evaluated the three genotypes of the 14‐base pair (bp) insertion/deletion polymorphism and reported that individuals carrying the homozygous del/del genotype exhibited a greater tendency toward HLA‐G expression, as assessed by immunohistochemistry, with staining scores ≥2 when compared with ins/ins carriers. Although this association did not reach statistical significance, the ins/ins genotype was consistently associated with lower HLA‐G expression, suggesting a potential regulatory effect of this polymorphism on protein expression levels [61].

Corroborating the association between the del allele and adverse clinical features, another study evaluating the 14 bp polymorphism in 150 patients diagnosed with NHL demonstrated a markedly increased risk of disease among individuals carrying the del/del (11.01‐fold) and ins/del (10.55‐fold) genotypes. The risk was higher in carriers of the del allele compared with those harboring the ins allele. Notably, ~30% of patients who experienced relapse during treatment carried either the del/del or ins/del genotypes, with poorer outcomes observed among del/del carriers. No significant associations were detected between HLA‐G genotypes and demographic variables or hepatitis C virus seropositivity; however, the del/del and ins/del genotypes were associated with hypertension in the case group, highlighting potential links between HLA‐G‐mediated immunoregulation and systemic comorbidities [63].

The relevance of sHLA‐G in lymphoproliferative disorders was further demonstrated by Sebti et al., who quantified plasma sHLA‐G levels in 103 patients with hematological malignancies, including B‐cell NHL (*n* = 75, *p* <0.001), T‐cell NHL (*n* = 11, *p* <0.005), and CLL (*n* = 17, *p* <0.001), compared with 30 healthy controls. Patients exhibited higher sHLA‐G concentrations across all disease groups, with mean levels of 46.42 ± 29.95 ng/mL in B‐NHL, 45.78 ± 48.66 ng/mL in T‐NHL, and 51.47 ± 23.56 ng/mL in CLL, compared with 18.57 ± 9.29 ng/mL in controls. These findings support the concept that elevated circulating HLA‐G represents a common feature of lymphoid malignancies and may contribute to systemic immune suppression [64].

**Figure 3:**
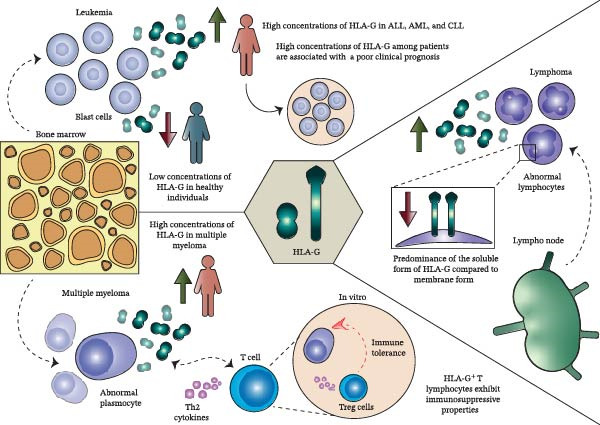
HLA‐G expression and immunological impact in hematological malignancies. Elevated levels of HLA‐G are observed in patients with acute lymphoblastic leukemia (ALL), acute myeloid leukemia (AML), lymphomas, and multiple myeloma (MM), in contrast to the low levels detected in healthy individuals. Increased HLA‐G expression is associated with poor clinical outcomes. In leukemias, HLA‐G is linked to the presence of blast cells in the bone marrow, whereas in MM it is associated with malignant plasma cells. In the context of MM, in vitro studies have shown that HLA‐G^+^ T lymphocytes exhibit immunosuppressive properties, contributing to immune tolerance through the induction of regulatory T cells (Tregs) and Th2‐type cytokines responses. Notably, the soluble form of HLA‐G predominates over the membrane‐bound form within the tumor microenvironment. Abbreviations: ALL, acute lymphoblastic leukemia; AML, acute myeloid leukemia; MM, multiple myeloma.

Additional studies assessing HLA‐G expression in lymphoproliferative diseases have suggested a predominance of the soluble form over membrane‐bound expression (Figure 3). In an analysis of 300 patients with B‐NHL, T‐NHL, immature NHL, and B‐CLL, soluble HLA‐G transcripts were detected in the majority of samples analyzed, whereas flow cytometry and immunohistochemical assays using the monoclonal antibody 87G failed to detect intracellular or surface HLA‐G expression. However, the limited number of samples analyzed for mRNA and surface protein expression restricted the ability to correlate these findings with clinical outcomes, underscoring the need for larger, well‐characterized cohorts [65].

In cutaneous lymphomas, HLA‐G expression has been detected in malignant cells from select subtypes. Immunoreactivity to HLA‐G was observed in CD8^+^ lymphomas and in a subset of CD56^+^ and CD4^+^ cases, frequently accompanied by local expression of the immunoregulatory cytokine interleukin‐10 (IL‐10). Moreover, expression of the inhibitory receptor ILT2 in large blastoid lymphocytes co‐localized with HLA‐G immunoreactivity, suggesting a coordinated immunosuppressive signaling axis within the tumor microenvironment [66].

In DLBCL, immunohistochemical analysis of diagnostic samples from 148 patients revealed HLA‐G expression in ~24% of cases. Interestingly, negative HLA‐G expression at diagnosis was associated with a lower likelihood of achieving complete remission following first‐line therapy. Furthermore, patients with HLA‐G‐positive tumors exhibited a 3‐year improvement in overall survival compared with those lacking HLA‐G expression, although this association did not reach statistical significance. These findings highlight the complex and context‐dependent role of HLA‐G in lymphoma biology, where its expression may reflect distinct immune–tumor interactions rather than uniformly adverse prognostic implications [67].

### 4.3. Multiple Myeloma

Consistent with the immunomodulatory and tolerogenic properties attributed to HLA‐G molecules, MM represents a paradigmatic example of how malignant cells exploit immune inhibitory pathways to evade cytotoxic responses. In a study investigating resistance to NK cell‐mediated cytotoxicity, both MM and CLL tumor cells were shown to utilize membrane‐bound HLA‐G1 as a mechanism to impair NK cell recognition and activation. Bone marrow aspirates from 13 MM patients and peripheral blood samples from 19 CLL patients were analyzed, with healthy individuals serving as controls. These findings demonstrated that HLA‐G‐mediated immune escape operates across distinct hematological malignancies and anatomical compartments, particularly within the bone marrow microenvironment [68].

Beyond direct receptor‐mediated inhibition, additional tumor‐promoting mechanisms involving HLA‐G have been described in MM. One such mechanism is trogocytosis, a process by which malignant plasma cells transfer membrane‐bound HLA‐G molecules to neighboring immune cells. In a cohort of 56 MM patients, increased expression of HLA‐G‐positive T lymphocytes was observed when compared with healthy individuals. Notably, the frequency of HLA‐G^+^ T cells was significantly higher in bone marrow samples (1.25%) than in peripheral blood (0.5%). Functional assays further demonstrated that HLA‐G^+^ T lymphocytes acquired immunosuppressive properties in vitro, resembling regulatory T cell‐like activity and contributing to the attenuation of antitumor immune responses [69]. These observations underscore the capacity of MM cells to actively reshape the immune landscape through both direct and indirect HLA‐G‐dependent mechanisms.

### 4.4. HLA‐G In Immunotherapy

In pathological contexts, particularly cancer, this same tolerogenic capacity is hijacked by tumor cells to facilitate immune evasion. As a result, HLA‐G has gained increasing attention in the field of cancer immunotherapy as a nonclassical immune checkpoint molecule, frequently associated with disease progression and unfavorable clinical outcomes [70–73].

Tumor cells capable of producing high levels of membrane‐bound or soluble HLA‐G can effectively suppress cytotoxic immune responses, thereby escaping immune surveillance. From a therapeutic perspective, strategies targeting HLA‐G‐mediated immune inhibition have been proposed, including (i) the development of blocking antibodies directed against HLA‐G or its inhibitory receptors and (ii) combination approaches involving neutralizing antibodies targeting classical immune checkpoints, with the goal of restoring effective antitumor immunity [74, 75].

To date, no clinical trials specifically targeting HLA‐G have been conducted in patients with hematological malignancies. However, several clinical studies are currently underway in the context of solid tumors, highlighting the translational potential of this pathway (Table 1). Among these, TTX‐080, an antagonist of HLA‐G receptor signaling, is being evaluated as monotherapy or in combination with established immune checkpoint inhibitors such as pembrolizumab, a programmed cell death protein 1 (PD‐1) inhibitor. Additionally, combination strategies involving agents such as cetuximab, a chimeric monoclonal antibody targeting the epidermal growth factor receptor (EGFR), aim to synergistically enhance CTL‐mediated antitumor activity [76].

**Table 1:** Ongoing and completed clinical studies targeting the HLA‐G pathway in solid tumors.

Medicines	Type of cancer	Mechanism of action	Onset	Clinical phase	Status	Study identifier
TTX‐080	HNSCC, NSCLC, colorectal cancer, triple negative breast cancer, renal cell carcinoma and acral melanoma	HLA‐G receptor antagonists	July, 2020	1	Not recruiting	NCT04485013
JNJ‐78306358	Solid tumors in advanced stages	Biospecific CD3 ligand on T cells and HLA‐G on cancer cells	October, 2021	1	Finished	NCT04991740
IVS‐3001	Previously treated, locally advanced or metastatic solid tumors that are HLA‐G positive (HLA‐G+)	Autologous CAR‐T cells	June, 2023	1/2a	Recruiting	NCT05672459
RO7515629	Advanced or metastatic solid tumors expressing HLA‐G	—	June, 2023	1	Recruiting	NCT05769959

*Note:* Summary of clinical trials evaluating therapeutic strategies directed at HLA‐G or HLA‐G–related pathways in solid malignancies, including monotherapy and combination approaches with immune checkpoint inhibitors.

Abbreviations: HNSCC, head and neck squamous cell carcinoma; NSCLC, non–small cell lung cancer.

Beyond receptor blockade, innovative immunotherapeutic strategies exploiting HLA‐G expression have also been developed. JNJ‐78306358, a bispecific molecule targeting CD3 on T cells and HLA‐G on tumor cells, has demonstrated the ability to redirect CTLs toward HLA‐G‐expressing malignant cells. Preclinical studies showed potent and specific antitumor activity in vitro and in vivo, with HLA‐G‐dependent cytotoxicity observed in murine models (81,82). However, early phase I clinical studies evaluating safety and tolerability reported dose‐limiting toxicities and the development of anti‐drug antibodies, precluding dose escalation and progression to phase II trials [77].

The restricted expression of HLA‐G in normal tissues, coupled with its frequent overexpression in tumor cells, has further positioned this molecule as an attractive target for chimeric antigen receptor (CAR)‐based therapies. In this context, autologous anti‐HLA‐G CAR‐T cells (IVS‐3001) are currently under investigation for the treatment of advanced‐stage HLA‐G–positive tumors. Preclinical in vivo studies demonstrated robust antitumor efficacy, with treated animals exhibiting near‐complete suppression of tumor growth [78, 79]. Phase I/IIa clinical trials are ongoing to assess the safety, tolerability, and long‐term efficacy of this approach in cancer patients.

In addition to its relevance in cancer immunotherapy, HLA‐G plays a critical role in transplantation tolerance. Elevated HLA‐G expression following allogeneic hematopoietic stem cell transplantation has been associated with reduced severity of donor lymphocyte‐mediated host reactions and improved transplant outcomes [80, 81]. Conversely, low circulating levels of soluble HLA‐G have been linked to an increased risk of acute graft‐versus‐host disease (aGvHD), underscoring its immunoregulatory importance in post‐transplant immune homeostasis [82].

Despite promising advances in solid tumors, the role of HLA‐G‐targeted immunotherapy in hematological malignancies remains largely unexplored. Given the profound immunological complexity of the bone marrow and lymphoid microenvironments, further studies are urgently needed to elucidate the expression patterns of HLA‐G in malignant and nonmalignant cells, as well as the impact of HLA‐G‐directed therapies administered alone or in combination with other immune modulators. Such investigations will be essential to determine the therapeutic potential of targeting HLA‐G in hematological cancers and to optimize immunotherapeutic strategies aimed at improving patient outcomes.

### 4.5. Conclusion and Perspectives

Although HLA‐G plays a fundamental physiological role in maintaining immune tolerance in selected tissues, its tight regulation during tumorigenesis and its functional similarities to tumor‐associated antigens and immune checkpoints position this molecule as a critical mediator of cancer immune evasion. The aberrant expression of HLA‐G in hematological malignancies highlights its relevance for understanding how tumor cells manipulate immune responses to escape immunosurveillance and underscores its potential as a therapeutic target capable of complementing existing cancer treatments.

Importantly, deeper insight into the biological mechanisms underlying HLA‐G expression and function in hematological malignancies may inform the development of novel immunotherapeutic strategies, enhance treatment responses, and facilitate the identification of clinically relevant prognostic biomarkers. Although HLA‐G has not yet been comprehensively investigated across leukemias, lymphomas, and MM, the studies available to date consistently indicate that its expression is positively regulated through multiple, often overlapping mechanisms. Despite this growing body of evidence, the precise contribution of HLA‐G to immune resistance and systemic immunosuppression in hematological tumors remains insufficiently characterized.

There is therefore a clear need for detailed mechanistic studies addressing how HLA‐G modulates antitumor immune responses, particularly those mediated by cytotoxic CD8^+^ T lymphocytes and natural killer cells. In addition, further investigation is warranted to clarify its role in leukemic cell trafficking, tissue infiltration, and disease dissemination within hematopoietic and lymphoid niches. Addressing these gaps will be essential to fully elucidate the functional relevance of HLA‐G in hematological cancer biology.

From a translational perspective, advances in immunotherapy provide a promising framework for incorporating HLA‐G‐targeted approaches into existing treatment paradigms. Novel combinatorial strategies may include immune checkpoint blockade regimens that integrate inhibition of the HLA‐G axis alongside established targets. Moreover, adoptive cell therapies, such as chimeric antigen receptor (CAR)‐T cells engineered to recognize HLA‐G‐expressing malignant cells, represent an innovative avenue for reinvigorating antitumor immunity in both autologous and allogeneic settings.

Ultimately, only continued and rigorous investigation will define the true clinical and prognostic impact of HLA‐G in hematological malignancies and determine whether emerging therapeutic approaches currently under evaluation will translate into meaningful patient benefit. The implications of these efforts, however, extend beyond oncology, as insights gained from HLA‐G‐mediated immune regulation may also inform broader aspects of immunosurveillance in infection, autoimmunity, and transplantation biology.

## Funding

This study was supported by Fundação de Amparo à Pesquisa do Estado do Amazonas (PRÓ‐ESTADO Program— Grants #002/2008, #007/2018, and #005/2019 and POSGRAD Program— Grants #002/2025 and #015/2026) the Genomic Surveillance Network in Health of the State of Amazonas, the Conselho Nacional de Desenvolvimento Científico e Tecnológico (INCT‐Sangue—Process #405918/2022‐4), the Coordenação de Aperfeiçoamento de Pessoal de Nível Superior (PDPG‐CONSOLIDACAO‐3‐4 Program— Process #88887.707248/2022‐0).

## Disclosure

The funders had no role in study design, decision to publish, or preparation of the manuscript.

## Conflicts of Interest

The authors declare no conflicts of interest.

## Data Availability

Data sharing is not applicable to this article as no datasets were generated or analyzed during the current study.

## References

[1] Taylor J. , Xiao W. , and Abdel-Wahab O. , Diagnosis and Classification of Hematologic malignancies on the Basis of Genetics, Blood. (2017) 130, no. 4, 410–423, 10.1182/blood-2017-02-734541, 2-s2.0-85026309285. 28600336 PMC5533199

[2] Whiteley A. E. , Price T. T. , Cantelli G. , and Sipkins D. A. , Leukaemia: A Model Metastatic Disease, Nature Reviews Cancer. (2021) 21, no. 7, 461–475, 10.1038/s41568-021-00355-z. 33953370 PMC8722462

[3] Thandra K. C. , Barsouk A. , Saginala K. , Padala S. A. , Barsouk A. , and Rawla P. , Epidemiology of non-Hodgkin’s Lymphoma, Medical Sciences. (2021) 9, no. 1, 10.3390/medsci9010005, 5. 33573146 PMC7930980

[4] Zanoni L. , Bezzi D. , and Nanni C. , et al.PET/CT in Non-Hodgkin Lymphoma: An Update, Seminars in Nuclear Medicine. (2023) 53, no. 3, 320–351, 10.1053/j.semnuclmed.2022.11.001. 36522191

[5] Firth J. , Haematology: Multiple Myeloma, Clinical Medicine. (2019) 19, no. 1, 58–60, 10.7861/CLINMEDICINE.19-1-58, 2-s2.0-85060137264. 30651246 PMC6399642

[6] Minnie S. A. and Hill G. R. , Immunotherapy of Multiple Myeloma, Journal of Clinical Investigation. (2020) 130, no. 4, 1565–1575, 10.1172/JCI129205. 32149732 PMC7108923

[7] Huang J. , Huang X. , and Huang J. , CAR-T Cell Therapy for Hematological malignancies: Limitations and Optimization Strategies, Frontiers in immunology. (2022) 13, 1019115. 36248810 10.3389/fimmu.2022.1019115 PMC9557333

[8] Ferreira L. M. R. , Meissner T. B. , Tilburgs T. , and Strominger J. L. , HLA-G: At the Interface of Maternal–Fetal Tolerance, Trends in Immunology. (2017) 38, no. 4, 272–286, 10.1016/J.IT.2017.01.009, 2-s2.0-85014448693. 28279591

[9] Tronik-Le Roux D. , Renard J. , and Vérine J. , et al.Novel Landscape of HLA-G Isoforms Expressed in Clear Cell Renal Cell Carcinoma Patients, Molecular Oncology. (2017) 11, no. 11, 1561–1578, 10.1002/1878-0261.12119, 2-s2.0-85029387974. 28815885 PMC5664004

[10] Hauer V. , Risti M. , and Miranda B. L. M. , et al.The Association of HLA-G Polymorphisms and the Synergistic Effect of sMICA and sHLA-G With Chronic Kidney Disease and Allograft Acceptance, PLOS ONE. (2019) 14, no. 2, 10.1371/journal.pone.0212750, 2-s2.0-85061990871. PMC6386361 30794652

[11] dos Almeida R. S. , de Ramos A. M. L. , Luna C. F. , Pedrosa F. , Donadi E. A. , and Lucena-Silva N. , Cytokines and Soluble HLA-G Levels in Bone Marrow Stroma and Their Association With the Survival Rate of Patients Exhibiting Childhood T-Cell Acute Lymphoblastic Leukemia, Cytokine. (2018) 102, no. 102, 94–101, 10.1016/j.cyto.2017.07.014, 2-s2.0-85028315309. 28802664

[12] Kirszenbaum M. , Moreau P. , Gluckman E. , Dausset J. , and Carosella E. , An Alternatively Spliced Form of HLA-G mRNA in Human Trophoblasts and Evidence for the Presence of HLA-G Transcript in Adult Lymphocytes, Proceedings of the National Academy of Sciences. (1994) 91, no. 10, 4209–4213, 10.1073/pnas.91.10.4209, 2-s2.0-0028346019. PMC43754 8183892

[13] Rizzo R. , Audrito V. , and Vacca P. , et al.HLA-G Is a Component of the Chronic Lymphocytic Leukemia Escape Repertoire to Generate Immune Suppression: Impact of the HLA-G. 14 Base Pair (rs66554220) Polymorphism, Haematologica. (2014) 99, no. 5, 888–896, 10.3324/haematol.2013.095281, 2-s2.0-84899752925. 24362551 PMC4008099

[14] Carosella E. D. , Dausset J. , and Rouas-Freiss N. , Immunotolerant Functions of HLA-G, Cellular and Molecular Life Sciences. (1999) 55, no. 3, 327–333, 10.1007/s000180050295, 2-s2.0-0032896987. 10228553 PMC11146997

[15] Dias F. C. , Castelli E. C. , Collares C. V. A. , Moreau P. , and Donadi E. A. , The Role of HLA-G Molecule and HLA-G Gene Polymorphisms in Tumors, Viral Hepatitis, and Parasitic Diseases, Frontiers in Immunology. (2015) 6, no. FEB, 2–11, 10.3389/fimmu.2015.00009, 2-s2.0-84926641260. 25699038 PMC4313582

[16] Gomes R. P. , Caetano G. T. , and Sampaio L. H. F. , et al.Evaluation of HLA-G Immune Checkpoint Molecule in Melanocytic Lesions, Jornal Brasileiro de Patologia e Medicina Laboratorial. (2020) 56, 1–4, 10.5935/1676-2444.20200016.

[17] Amiot L. , Le Friec G. , and Sebti Y. , et al.HLA-G and Lymphoproliferative Disorders, Seminars in Cancer Biology. (2003) 13, no. 5, 379–385, 10.1016/S1044-579X(03)00029-4, 2-s2.0-10744231744. 14708718

[18] Young N. S. , Acquired Aplastic Anemia, Annals of Internal Medicine. (2002) 923, no. 34, 10.1016/B978-0-12-812102-3.00049-X. 11926789

[19] Galanello R. and Origa R. , Beta-Thalassemia, Orphanet Journal of Rare Diseases. (2010) 5, no. 1, 1–15, 10.1186/1750-1172-5-11, 2-s2.0-77952432110. 20492708 PMC2893117

[20] Baxter E. J. , Scott L. M. , and Campbell P. J. , et al.Acquired Mutation of the Tyrosine Kinase JAK2 in Human Myeloproliferative Disorders, The Lancet. (2005) 365, no. 9464, 1054–1061, 10.1016/s0140-6736(05)71142-9. 15781101

[21] James C. , Ugo V. R. , and Le Coué J. P. , et al.A Unique Clonal JAK2 Mutation Leading to Constitutive Signalling Causes Polycythaemia Vera, Nature. (2005) 434, no. 7037, 1144–1148, 10.1038/nature03546, 2-s2.0-17844383458. 15793561

[22] Nangalia J. and Green A. R. , Myeloproliferative Neoplasms: From Origins to Outcomes, Blood. (2017) 2017, no. 1, 470–479, 10.1182/asheducation-2017.1.470, 2-s2.0-85038429444. 29212804

[23] Marisabel H. C. , Brian F. , and Morris J. , Immune Thrombocytopenic Purpura With Subsequent Development of JAK2 V617F-Positive Essential Thrombocythemia: Case Report, Archives of Hematology Case Reports and Reviews. (2021) 6, 018–020, 10.17352/ahcrr.000033.

[24] Menier C. , Carosella E. , and Rouas-Freiss N. , Use of Compositions Comprising a Soluble Form of Hla-g in the Treatment of Blood Diseases, 2004, United States;US20070020703A1.

[25] HoWangYin K. Y. , Loustau M. , and Wu J. , et al.Multimeric Structures of HLA-G Isoforms Function Through Differential Binding to LILRB Receptors, Cellular and Molecular Life Sciences. (2012) 69, no. 23, 4041–4049, 10.1007/s00018-012-1069-3, 2-s2.0-84869065865. 22802125 PMC3884069

[26] Paul P. , Adrian Cabestre F. , and Ibrahim E. C. , et al.Identification of HLA-G7 as a New Splice Variant of the HLA-G mRNA and Expression of Soluble HLA-G5, -G6, and -G7 Transcripts in Human Transfected Cells, Human Immunology. (2000) 61, no. 11, 1138–1149, 10.1016/S0198-8859(00)00197-X, 2-s2.0-0034516969. 11137219

[27] Rudstein-Svetlicky N. , Loewenthal R. , Horejsi V. , and Gazit E. , HLA-G Levels in Serum and Plasma, Tissue Antigens. 67, no. 2. 10.1111/j.1399-0039.2006.00540.x 16441481

[28] Colonna M. , Navarro F. , and Bellón T. , et al.A Common Inhibitory Receptor for Major Histocompatibility Complex Class I Molecules on Human Lymphoid and Myelomonocytic Cells, The Journal of Experimental Medicine. (1997) 186, no. 11, 1809–1818, 10.1084/jem.186.11.1809, 2-s2.0-0030819308. 9382880 PMC2199153

[29] Gros F. , Sebti Y. , and De Guibert S. , et al.Soluble HLA-G Molecules Are Increased During Acute Leukemia, Especially in Subtypes Affecting Monocytic and Lymphoid Lineages’, Neoplasia. (2006) 8, no. 3, 223–230, 10.1593/neo.05703, 2-s2.0-33646339671. 16611416 PMC1578523

[30] Faure M. and Long E. O. , KIR2DL4 (CD158d), an NK Cell-Activating Receptor With Inhibitory Potential, The Journal of Immunology. (2002) 168, no. 12, 6208–6214, 10.4049/jimmunol.168.12.6208, 2-s2.0-0037097664. 12055234

[31] Carosella E. D. , Moreau P. , LeMaoult J. , and Rouas-Freiss N. , HLA-G: From Biology to Clinical Benefits, Trends in Immunology. (2008) 29, no. 3, 125–132, 10.1016/j.it.2007.11.005, 2-s2.0-39949083940. 18249584

[32] Du L. , Xiao X. , and Wang C. , et al.Human Leukocyte Antigen-G Is Closely Associated With Tumor Immune Escape in Gastric Cancer by Increasing Local Regulatory T Cells, Cancer Science. (2011) 102, no. 7, 1272–1280, 10.1111/j.1349-7006.2011.01951.x, 2-s2.0-79959217991. 21466615

[33] Bernstein C. N. , Blanchard J. F. , Kliewer E. , and Wajda A. , Cancer Risk in Patients With Inflammatory Bowel Disease, Cancer. (2001) 91, no. 4, 854–862. 11241255 10.1002/1097-0142(20010215)91:4<854::aid-cncr1073>3.0.co;2-z

[34] Okumura T. , Joshita S. , and Yamazaki T. , et al.HLA-G Susceptibility to Hepatitis B Infection and Related Hepatocellular Carcinoma in the Japanese Population, Human Immunology. (2023) 84, no. 8, 401–407, 10.1016/j.humimm.2023.05.002. 37271588

[35] Zambra F. M. B. , Biolchi V. , De Cerqueira C. C. S. , Brum I. S. , Castelli E. C. , and Chies J. A. B. , Immunogenetics of Prostate Cancer and Benign Hyperplasia – the Potential use of an Hla-g Variant as a Tag Snp for Prostate Cancer Risk, HLA. (2016) 87, no. 2, 79–88, 10.1111/tan.12741, 2-s2.0-85010727680. 26889902

[36] Zheng G. , Jia L. , and Yang A. G. , Roles of HLA-G/KIR2DL4 in Breast Cancer Immune Microenvironment, Frontiers in Immunology. (2022) 13, 1–8, 10.3389/fimmu.2022.791975. PMC8850630 35185887

[37] Cao M. , Yie S. M. , Liu J. , Ye S. R. , Xia D. , and Gao E. , Plasma Soluble HLA-G Is a Potential Biomarker for Diagnosis of Colorectal, Gastric, Esophageal and Lung Cancer, Tissue Antigens. (2011) 78, no. 2, 120–128, 10.1111/j.1399-0039.2011.01716.x, 2-s2.0-79959928969. 21726203

[38] Lin A. and Yan W. H. , Heterogeneity of HLA-G Expression in Cancers: Facing the Challenges, Frontiers in Immunology. (2018) 9, 395508. 10.3389/fimmu.2018.02164 PMC6170620 30319626

[39] Pistoia V. , Morandi F. , Wang X. , and Ferrone S. , Souble HLA-G Are they Clinically Relevant?, Seminars in Cancer Biology. (2007) 17, no. 6, 469–479, 10.1016/j.semcancer.2007.07.004, 2-s2.0-35948961915. 17825579 PMC2200630

[40] Scarabel L. , Garziera M. , Fortuna S. , Asaro F. , Toffoli G. , and Geremia S. , Soluble HLA-G Expression Levels and HLA-G/Irinotecan Association in Metastatic Colorectal Cancer Treated With Irinotecan-Based Strategy, Scientific Reports. (2020) 10, no. 1, 1–11, 10.1038/s41598-020-65424-z. 32471996 PMC7260212

[41] Le Gal F. A. , Riteau B. , and Sedlik C. , et al.HLA-G-Mediated Inhibition of Antigen-Specific Cytotoxic T Lymphocytes, International Immunology. (1999) 11, no. 8, 1351–1356, 10.1093/intimm/11.8.1351, 2-s2.0-0032788734. 10421792

[42] Terzieva A. , Alexandrova M. , Manchorova D. , Slavov S. , Djerov L. , and Dimova T. , HLA-G Expression/Secretion and T-Cell Cytotoxicity in Missed Abortion in Comparison to Normal Pregnancy, International Journal of Molecular Sciences. (2024) 25, no. 5, 10.3390/ijms25052643, 2643. 38473890 PMC10932117

[43] Lin A. and Yan W. H. , Perspective of HLA-G Induced Immunosuppression in SARS-CoV-2 Infection, Frontiers in Immunology. (2021) 12, 10.3389/fimmu.2021.788769, 788769. 34938296 PMC8685204

[44] Carosella E. D. , Gregori S. , and LeMaoult J. , The Tolerogenic Interplay(s) Among HLA-G, Myeloid APCs, and Regulatory Cells, Blood. (2011) 118, no. 25, 6499–6505, 10.1182/blood-2011-07-370742, 2-s2.0-84055218980. 21960588

[45] LeMaoult J. , Krawice-Radanne I. , Dausset J. , and Carosella E. D. , HLA-G1-Expressing Antigen-Presenting Cells Induce Immunosuppressive CD4+ T Cells, Proceedings of the National Academy of Sciences. (2004) 101, no. 18, 7064–7069, 10.1073/pnas.0401922101, 2-s2.0-2342559752. PMC406466 15103024

[46] Bu X. , Zhong J. , Li W. , Cai S. , Gao Y. , and Ping B. , Immunomodulating Functions of Human Leukocyte Antigen-G and Its Role in Graft-Versus-Host Disease after Allogeneic Hematopoietic Stem Cell Transplantation, Annals of Hematology. (2021) 100, no. 6, 10.1007/s00277-021-04486-z, 1391. 33709198 PMC8116272

[47] Naji A. , Durrbach A. , Carosella E. D. , and Rouas-Freiss N. , Soluble HLA-G and HLA-G1 Expressing Antigen-Presenting Cells Inhibit T-Cell Alloproliferation Through ILT-2/ILT-4/FasL-Mediated Pathways, Human Immunology. (2007) 68, no. 4, 233–239, 10.1016/j.humimm.2006.10.017, 2-s2.0-33947657929. 17400057

[48] Lucas D. , The Bone Marrow Microenvironment for Hematopoietic Stem Cells, Advances in Experimental Medicine and Biology. (2017) 1041, 5–18, 10.1007/978-3-319-69194-7_2, 2-s2.0-85042486890. 29204826

[49] Wobus M. and Bornhäuser M. , Deciphering the Bone Marrow Microenvironment in Hematologic Malignancies, Frontiers in Oncology. (2023) 13, 10.3389/fonc.2023.1231467, 1231467. 37404763 PMC10316019

[50] Mian S. A. , Ngo S. , and Bonnet D. , Is the Bone Marrow Microenvironment the Hidden Catalyst in Malignant Haematopoiesis?, Leukemia. (2025) 39, no. 7, 1589–1592, 10.1038/s41375-025-02630-6. 40301617 PMC12208878

[51] Mizuno S. , Emi N. , Kasai M. , Ishitani A. , and Saito H. , Aberrant Expression of HLA-G Antigen in Interferon γ-Stimulated Acute Myelogenous Leukaemia, British Journal of Haematology. (2000) 111, no. 1, 280–282, 10.1111/j.1365-2141.2000.02345.x. 11091213

[52] Nückel H. , Rebmann V. , Dürig J. , Dührsen U. , and Grosse-Wilde H. , HLA-G Expression Is Associated With an Unfavorable Outcome and Immunodeficiency in Chronic Lymphocytic Leukemia, Blood. (2005) 105, no. 4, 1694–1698, 10.1182/blood-2004-08-3335, 2-s2.0-13544272581. 15466928

[53] Locafaro G. , Amodio G. , Tomasoni D. , Tresoldi C. , Ciceri F. , and Gregori S. , HLA-G Expression on Blasts and Tolerogenic Cells in Patients Affected by Acute Myeloid Leukemia, Journal of Immunology Research. (2014) 2014. 10.1155/2014/636292 PMC3987970 24741612

[54] Arnaiz-Villena A. , Juarez I. , and Suarez-Trujillo F. , et al.HLA-G: Function, Polymorphisms and Pathology, International Journal of Immunogenetics. (2021) 48, no. 2, 172–192, 10.1111/iji.12513. 33001562

[55] Rezvany M. R. , Kazemi A. , Hajifathali A. , Kaviani S. , and Mellstedt H. , Analysis of HLA-G Gene Expression in B-Lymphocytes From Chronic Lymphocytic Leukemia Patients, Iranian Biomedical Journal. (2007) 11, no. 2, 125–129. 18051955

[56] Caocci G. , Greco M. , and Arras M. , et al.HLA-G Molecules and Clinical Outcome in Chronic Myeloid Leukemia, Leukemia Research. (2017) 61, 1–5, 10.1016/j.leukres.2017.08.005, 2-s2.0-85027848700. 28841441

[57] Castelli E. C. , Mendes-Junior C. T. , and Deghaide N. H. S. , et al.The Genetic Structure of 3′untranslated Region of the HLA-G Gene: Polymorphisms and Haplotypes, Genes & Immunity. (2010) 11, no. 2, 134–141, 10.1038/gene.2009.74, 2-s2.0-77649335964. 19798077

[58] de Almeida B. S. , Muniz YCN. , Prompt A. H. , Castelli E. C. , Mendes-Junior C. T. , and Donadi E. A. , Genetic Association Between HLA-G. 14-Bp Polymorphism and Diseases: A Systematic Review and Meta-Analysis, Human Immunology. (2018) 79, no. 10, 724–735, PubMed 10.1016/j.humimm.2018.08.003, 2-s2.0-85051392178. 30102938

[59] Chen D. P. , Wang P. N. , and Hour A. L. , et al.The Association Between Genetic Variants at 3^′^-UTR and 5^′^-URR of HLA-G Gene and the Clinical Outcomes of Patients With Leukemia Receiving Hematopoietic Stem Cell Transplantation, Frontiers in Immunology. (2023) 14, 10.3389/fimmu.2023.1093514. PMC9995383 36911734

[60] Gros F. , Cabillic F. , Tourirais O. , Le Maux A. , Sebti Y. , and Amiot L. , Soluble HLA-G Molecules Impair Natural Killer/Dendritic Cell Crosstalk via Inhibition of Dendritic Cells, European Journal of Immunology. (2008) 38, no. 3, 742–749, 10.1002/eji.200736918, 2-s2.0-43649104999. 18266268

[61] Caocci G. , Greco M. , and Fanni D. , et al.HLA-G Expression and Role in Advanced-Stage Classical Hodgkin Lymphoma, European Journal of Histochemistry. (2016) 60, no. 2, 10.4081/ejh.2016.2606, 2-s2.0-84963631035. PMC4933823 27349312

[62] Pereira E. R. , Jones D. , Jung K. , and Padera T. P. , The Lymph Node Microenvironment and Its Role in the Progression of Metastatic Cancer, Seminars in Cell & Developmental Biology. (2015) 38, 98–105, 10.1016/j.semcdb.2015.01.008, 2-s2.0-84928146002. 25620792 PMC4397158

[63] Tawfeek G. A. E. and Alhassanin S. , HLA-G Gene Polymorphism in Egyptian Patients With Non-Hodgkin Lymphoma and Its Clinical Outcome, Immunological Investigations. (2018) 47, no. 3, 315–325, 10.1080/08820139.2018.1430826, 2-s2.0-85041585291. 29388862

[64] Sebti Y. , Le Friec G. , and Pangault C. , et al.Soluble HLA-G Molecules Are Increased in Lymphoproliferative Disorders, Human Immunology. (2003) 64, no. 11, 1093–1101, 10.1016/j.humimm.2003.08.345, 2-s2.0-0242693966. 14602240

[65] Amiot L. , Ferrone S. , Grosse-Wilde H. , and Seliger B. , Biology of HLA-G in Cancer: A Candidate Molecule for Therapeutic Intervention?, Cellular and Molecular Life Sciences. (2011) 68, no. 3, 417–431, 10.1007/s00018-010-0583-4, 2-s2.0-79951549780. 21063893 PMC3426238

[66] Urosevic M. , Kamarashev J. , Burg G. , and Dummer R. , Primary Cutaneous CD8+ and CD56+ T-Cell Lymphomas Express HLA-G and Killer-Cell Inhibitory Ligand, ILT2, Blood. (2004) 103, no. 5, 1796–1798, 10.1182/blood-2003-10-3372, 2-s2.0-1442332337. 14592815

[67] Jesionek-Kupnicka D. , Bojo M. , and Prochorec-Sobieszek M. , et al.HLA-G and MHC Class II Protein Expression in Diffuse Large B-Cell Lymphoma, Archivum Immunologiae et Therapiae Experimentalis. (2016) 64, no. 3, 225–240, 10.1007/s00005-015-0372-8, 2-s2.0-84949789717. 26667793

[68] Maki G. , Hayes G. M. , and Naji A. , et al.NK Resistance of Tumor Cells From Multiple Myeloma and Chronic Lymphocytic Leukemia Patients: Implication of HLA-G, Leukemia. (2008) 22, no. 5, 998–1006, 10.1038/leu.2008.15, 2-s2.0-43749089136. 18288133

[69] Brown R. , Kabani K. , and Favaloro J. , et al.CD86+or HLA-G+ Can Be Transferred via Trogocytosis From Myeloma Cells to T Cells and Are Associated With Poor Prognosis, Blood. (2012) 120, no. 10, 2055–2063, 10.1182/blood-2012-03-416792, 2-s2.0-84866156002. 22705596

[70] Krijgsman D. , Roelands J. , Hendrickx W. , Bedognetti D. , and Kuppen P. J. K. , HLA-G: A New Immune Checkpoint in Cancer, International Journal of Molecular Sciences. (2020) 21, no. 12, 1–11, 10.3390/IJMS21124528. PMC7350262 32630545

[71] Carosella E. D. , Rouas-Freiss N. , Le Roux D. T. , Moreau P. , and LeMaoult J. , HLA-G: An Immune Checkpoint Molecule, Advances in immunology. (2015) 127, 33–144, 10.1016/BS.AI.2015.04.001, 2-s2.0-84945458642. 26073983

[72] Loustau M. , Anna F. , and Dréan R. , et al.HLA-G Neo-Expression on Tumors, Frontiers in Immunology. (2020) 11, 10.3389/FIMMU.2020.01685. PMC7456902 32922387

[73] Lin A. and Yan W. H. , HLA-G/ILTs Targeted Solid Cancer Immunotherapy: Opportunities and Challenges, Frontiers in Immunology. (2021) 12, 10.3389/FIMMU.2021.698677. PMC8278316 34276691

[74] Morandi F. and Airoldi I. , HLA-G and Other Immune Checkpoint Molecules as Targets for Novel Combined Immunotherapies, International Journal of Molecular Sciences. (2022) 23, no. 6, 10.3390/ijms23062925. PMC8948858 35328349

[75] Yan W. H. , HLA-G Expression in Cancers: Potential Role in Diagnosis, Prognosis and Therapy, Endocrine, Metabolic & Immune Disorders-Drug Targets. (2012) 11, no. 1, 76–89, 10.2174/187153011794982059, 2-s2.0-79953021083. 21348818

[76] Study Details | TTX-080 HLA-G Antagonist in Subjects With Advanced Cancers | ClinicalTrials.gov, 2023, https://clinicaltrials.gov/study/NCT04485013.

[77] Oh D. Y. , Lee E. , and Tolcher A. W. , et al.1045P Safety and Preliminary Clinical Activity of JNJ-78306358 (JNJ-358), an HLA-G and CD3 Bispecific Antibody, for the Treatment of Advanced Stage Solid Tumor, Annals of Oncology, Oct. (2023) 34, S633–S634, 10.1016/J.ANNONC.2023.09.2184.

[78] Anna F. , Bole-Richard E. , and Lemaoult J. , et al.First Immunotherapeutic CAR-T Cells Against the Immune Checkpoint Protein HLA-G, Journal for Immunotherapy of Cancer. (2021) 9, no. 3, 10.1136/JITC-2020-001998. PMC7978334 33737343

[79] A Safety And Efficacy Study Of HLA-G- Targeted CAR-T Cells IVS-3001 In Subjects With Previously Treated Advanced HLA-G-Positive Solid Tumors | ClinicalTrials.gov [Internet], 2023, https://clinicaltrials.gov/study/NCT05672459.

[80] González Á. , Rebmann V. , Lemaoult J. , Horn P. A. , Carosella E. D. , and Alegre E. , The Immunosuppressive Molecule HLA-G and Its Clinical Implications, Critical Reviews in Clinical Laboratory Sciences. (2012) 49, no. 3, 63–84, 10.3109/10408363.2012.677947, 2-s2.0-84861707985. 22537084

[81] Biedroń M. , Rybka J. , Wróbel T. , Prajs I. , Poręba R. , and Kuliczkowski K. , The Role of Soluble HLA-G and HLA-G Receptors in Patients With Hematological Malignancies After Allogeneic Stem Cell Transplantation, Medical Oncology. (2015) 32, no. 8, Aug 10.1007/S12032-015-0664-1, 2-s2.0-84938066032. 26187179

[82] Boukouaci W. , Busson M. , and Fortier C. , et al.Association of HLA-G Low Expressor Genotype With Severe Acute Graft-Versus-Host Disease After Sibling Bone Marrow Transplantation, Frontiers in Immunology. (2011) 2, 10.3389/FIMMU.2011.00074, 2-s2.0-84872122227. PMC3342264 22566863

[83] Connors J. M. , Cozen W. , and Steidl C. , et al.Hodgkin Lymphoma, Nature Reviews Disease Primers. (2020) 6, no. 1, 10.1038/s41572-020-0189-6, 61. 32703953

[84] Piris M. A. , Medeiros L. J. , and Chang K. C. , Hodgkin Lymphoma: A Review of Pathological Features and Recent Advances in Pathogenesis, Pathology, 2020, 52, no. 1, Elsevier, 154–165, 10.1016/j.pathol.2019.09.005. 31699300

[85] Martín-Villa J. M. , Vaquero-Yuste C. , and Molina-Alejandre M. , et al.HLA-G: Too Much or Too Little? Role in Cancer and Autoimmune Disease, Frontiers in Immunology. (2022) 10.3389/fimmu.2022.796054. PMC8829012 35154112

[86] Alkhouly N. , Shehata I. , Ahmed M. B. , Shehata H. , Hassan S. , and Ibrahim T. , HLA-G Expression in Acute Lymphoblastic Leukemia: A Significant Prognostic Tumor Biomarker, Medical Oncology. (2013) 30, no. 1, 10.1007/s12032-013-0460-8, 2-s2.0-84872271298. 23335072

[87] Obermajer N. , Zwolak A. , and Van De Ven K. , et al.Abstract ND07: JNJ-78306358: A First-in-Class Bispecific T Cell Redirecting HLA-G Antibody, Cancer Research. (2022) 82, no. 12_Supplement, ND07–ND07, 10.1158/1538-7445.AM2022-ND07.

[88] Study Details | A Study of JNJ-78306358 in Participants With Advanced Stage Solid Tumors | ClinicalTrials.gov [Internet], 2023, https://clinicaltrials.gov/study/NCT04991740.

